# Nanocrystalline SnO_2_:F Thin Films for Liquid Petroleum Gas Sensors

**DOI:** 10.3390/s110707127

**Published:** 2011-07-11

**Authors:** Sutichai Chaisitsak

**Affiliations:** Department of Electronics, Faculty of Engineering, King Mongkut’s Institute of Technology Ladkrabang, Bangkok 10520, Thailand; E-Mail: kcsutich@kmitl.ac.th; Tel.: +66-2-329-8344; Fax: +66-2-329-8346

**Keywords:** F-doped tin oxide films, dip-coating technique, liquid petroleum gas (LPG) sensors

## Abstract

This paper reports the improvement in the sensing performance of nanocrystalline SnO_2_-based liquid petroleum gas (LPG) sensors by doping with fluorine (F). Un-doped and F-doped tin oxide films were prepared on glass substrates by the dip-coating technique using a layer-by-layer deposition cycle (alternating between dip-coating a thin layer followed by a drying in air after each new layer). The results showed that this technique is superior to the conventional technique for both improving the film thickness uniformity and film transparency. The effect of F concentration on the structural, surface morphological and LPG sensing properties of the SnO_2_ films was investigated. Atomic Force Microscopy (AFM) and X-ray diffraction pattern measurements showed that the obtained thin films are nanocrystalline SnO_2_ with nanoscale-textured surfaces. Gas sensing characteristics (sensor response and response/recovery time) of the SnO_2_:F sensors based on a planar interdigital structure were investigated at different operating temperatures and at different LPG concentrations. The addition of fluorine to SnO_2_ was found to be advantageous for efficient detection of LPG gases, e.g., F-doped sensors are more stable at a low operating temperature (300 °C) with higher sensor response and faster response/recovery time, compared to un-doped sensor materials. The sensors based on SnO_2_:F films could detect LPG even at a low level of 25% LEL, showing the possibility of using this transparent material for LPG leak detection.

## 1. Introduction

Liquefied petroleum gas (LPG), which consists of hydrocarbons like CH_4_, C_3_H_8_, C_4_H_10_, *etc.*, is widely used for many domestic and industrial purposes as well as used as a fuel for automobiles. Since it is potentially explosive, the detection of gas leaks has become very important for preventing the occurrence of such accidents [1]. Among the metal oxides, tin oxide (SnO_2_) is one of the most widely used materials for gas sensor application because of its ease of fabrication and its special properties such as chemical and thermal stability, natural non-stoichiometry and good ability to absorb oxygen. However, the gas-sensing properties of pure SnO_2_ are not sufficient to identify a given gas, due to its low sensitivity and selectivity [2]. To improve sensor response and selectivity for LPG detection, until now, various additives [3], such as Pt [4], Pd [4–6], Si [7], Sb [8] and Cs [9] or their oxides have been incorporated into the SnO_2_. Grain size reduction is another approach to enhance the gas response [6,9–11], and various techniques have been developed to reduce the grain size. A brief review of the results on doped SnO_2_ sensors reported by several groups is summarized in Table 1. Fluorine (F) doped SnO_2_ coated on glasses are now widely developed as transparent and conduction substrates for use in optical and electronic applications [12]. According to the literature survey, however, there are very few reports available [13–15] on the developing SnO_2_:F thin-film based gas sensors. Several deposition techniques have been used to grow un-doped and doped SnO_2_ films, including sputtering [7], E-beam evaporation [4], spray pyrolysis [8,9] and sol-gel [6]. Among them, a dip-coating deposition [16] is one of the most promising ones, due to the simplicity of the apparatus, cost-effectiveness, good uniformity of the films and well suitability for large-scale production.

In this paper, we report the deposition of F-doped nanocrystalline SnO_2_ thin films using the dip-coating technique and their application in LPG sensors. The effect of F concentration on the structural, surface morphological and LPG sensing properties of the SnO_2_ films was studied. To the best of our knowledge, this is the first report on the LPG sensing properties of SnO_2_ films modified by F doping.

## 2. Experimental Section

### 2.1. Sample Preparations

A tin oxide film was prepared by using a homemade dip-coating apparatus, which consists of a precursor container, a step motor and a heater. The layer-by-layer deposition cycle was done by alternating between dip-coating a thin layer and drying in air after each new layer. The precursor used was 0.25 M of stannous chloride (SnCl_2_:2H_2_O; Carlo Erba) prepared in ethanol (C_2_H_5_OH; Carlo Erba). The fluorine doping was achieved using ammonium fluoride (NH_4_F; Merck). A slide glass was used as a substrate. The dopant concentration of the precursor (as wt.% of NH_4_F to SnCl_2_:2H_2_O) was varied from 0 to 15 wt.%. The mixed solution was stirred for 2∼3 h and followed by ultrasonic agitation for ∼30 min before usage. A cleaned substrate was dipped into the solution and withdrawn at a constant speed of 2.4 mm/min, then annealed in air at ∼400 °C for ∼30 s after each cycle (one cycle). The deposition cycles were completely automated by computerized control system. Un-doped and F-doped SnO_2_ sensors were fabricated using a planar interdigital structure (sensing area: 10 mm × 8 mm). A finger electrode of aluminum (thickness: ∼500 nm) was fabricated through shadow mask using evaporation technique under a vacuum pressure of 1 × 10^−3^ Torr.

### 2.2. Characterizations

XRD patterns of SnO_2_ films were obtained using CuKα radiation at 30 kV and 30 mA and the crystallite size was estimated by the Scherrer’s equation. The morphology of the films was observed by SEM and AFM. Optical and electrical properties of the films were examined in the 0.3 to 1.1 μm spectral range using a UV/VIS spectroscopy and Hall Effect measurement, respectively. The film thickness was measured by a step profilemeter. The fourier transform infrared (FTIR) measurement was conducted over the range of 400 to 4,000 cm^−1^ in transmission mode at room temperature. Most of the spectra in this work were averaged over three different positions on the samples.

A SnO_2_ thin film sensor with finger interdigital electrodes was mounted tightly on a heater in a homemade test chamber whose temperature could be measured and controlled by a thermocouple. The sensing characteristic was examined by monitoring the changes in resistance, with a constant voltage of 1 V using Keithley source meter (model 2004). The sensor response to LPG was defined as |*R_a_* *− R*_g_*|* × *100/R_a_*, where *R*_g_ is the sensor resistance in the presence of LPG and *R_a_* is that of baseline in N_2_ (or zero air) (TIG, Thailand; Industrial grade). The sensor response to 0.1∼6.4 vol.% of LPG was measured at 250∼400 °C. Commercially available LPG (PTT, Thailand) was used for this measurement. Varying LPG concentration was achieved by using a mass flow controller unit. The gas pressure over the sensor was 1 atm during the experiments. Data acquisition, storage and plotting in real time were realized using a personal computer with LabVIEW^®^ software via a GPIB interface control.

## 3. Results and Discussion

### 3.1. Thin Film Properties

**Figure 1.**
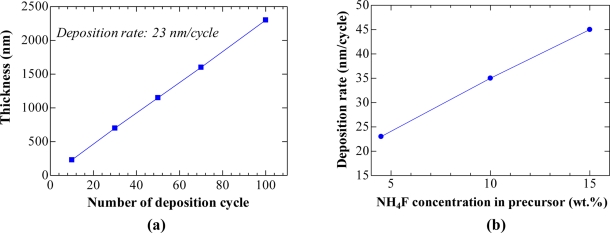
**(a)** Variation of film thickness with the number of deposition cycle (NH_4_F concentration: 4.5 wt.%). **(b)** Plot of deposition rate *vs.* NH_4_F concentration of the precursor.

The variation of film thickness with the number of deposition cycles in Figure 1(a) reveals that this technique is superior to the conventional technique for improving the film thickness uniformity. The desired thickness could be easily adjusted since the film thickness is nearly linear with the number of deposition cycle. For the 4.5 wt.% NH_4_F precursor [Figure 1(a)], the deposition rate was ∼23 nm/cycle. When concentration increased to 10 wt.% and 15 wt.%, the deposition rate increased to ∼35 and ∼ 45 nm/cycle respectively, as shown in Figure 1(b). The increase in deposition rate with increasing the NH_4_F concentration has been found previously in NH_4_F/SnCl_2_ spray pyrolysis [18].

**Figure 2.**
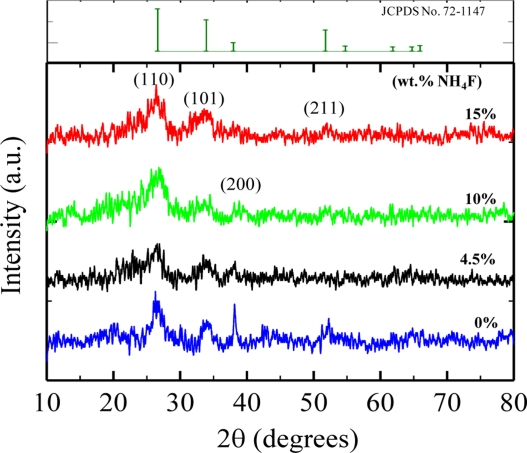
XRD patterns of SnO_2_ films deposited under different NH_4_F concentrations.

Figure 2 shows the X-ray diffraction patterns of the SnO_2_ films deposited at various NH_4_F concentrations (0, 4.5, 10, 15 at wt.%). For all deposited films, major peaks corresponding to the tetragonal SnO_2_ (JCPDS No. 72-1147) were observed.

**Figure 3.**
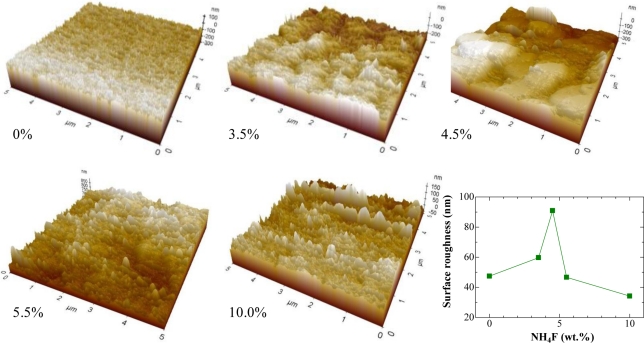
Three-dimensional AFM images of SnO_2_ films deposited under different NH_4_F concentrations. Inset: a plot of RMS roughness *vs.* NH_4_F concentration.

The peaks were broad, showing that the obtained films were small-sized nanocrystalline SnO_2._ The average crystalline size of SnO_2_ was estimated from XRD data using Scherrer’s equation [19] applied to the most intense 110, 101 and 200 diffraction lines. A broad size distribution ranging from 5.1 nm to 22.7 nm was found for the undoped film, however, the addition of NH_4_F into the precursor decreased the crystalline size and the size distribution became narrower (the calculated sizes were in the range of 3.7∼5.5 nm, 2.9∼3.9 nm and 2.7∼3.5 nm for 4.5 wt.%, 10 wt.% and 15 wt.%, respectively). Moreover, it was found that the crystalline size of films displayed very little dependence on the film thickness; the average size increased slightly from 4.3 nm for a 0.4 um-thick film to 5.0 nm for a 1.2 μm-thick film. Surface morphology of the films with different NH_4_F concentrations was examined by atomic force microscopy (AFM). The 3D images recorded at 5 μm × 5 m planar in contact mode are depicted in Figure 3.

**Figure 4.**
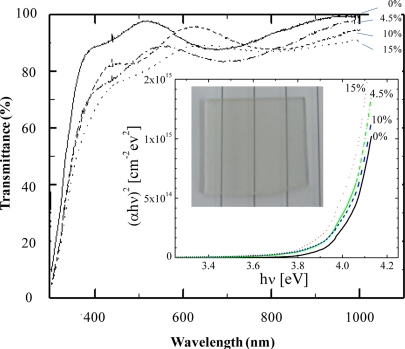
Transmittance spectra of SnO_2_ films deposited under different NH_4_F concentrations. Insets: plots of (αhν)^2^ *vs.* hν(photon energy) and photograph of 4.5 wt.% F-doped films (Area: ∼1 inch^2^).

Root mean square (RMS) roughness of the films was obtained from the AFM data (inset in Figure 3). It was clearly seen that the oxide thin films deposited by dip-coating technique showed a nanoscale texture. AFM study reveals that the roughness of the films is dependent on the doping concentration. As shown in the figure, the surface roughness increased with increasing NH_4_F concentration and reached a maximum of 90 nm at 4.5 wt.%. However, it decreased with further increases in concentration (>4.5 wt.%). The change in the surface roughness with varying NH_4_F concentration may be due to the deep/shadow distribution of fluorine atoms in the tin oxide structure [20] and also the vaporization of fluorine from the films during the annealing process [21]. Figure 4 shows the wavelength dependence on optical transmittance of SnO_2_:F thin films deposited with various NH_4_F concentrations. The transmittance of all samples was more than 70% in the whole visible-light region (*i.e.*, above 400 nm). The optical energy band gap (E_g_) calculated from the optical transmission [22] was in the range of 3.95∼4.05 eV, and was found to slightly decrease for higher NH_4_F dopant (inset in Figure 4). The obtained E_g_ in this work was higher than those reported in previous works [23,24]. This could be due to the small grain size effect of the films [25].

**Figure 5.**
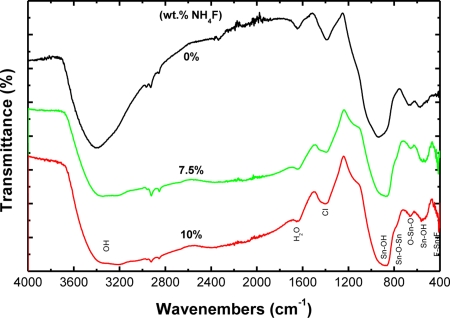
FTIR spectra of un-doped and F-doped SnO_2_ films.

FTIR spectrometry was used for the determination of existing surface species. The FTIR spectra of un-doped and F-doped SnO_2_ films are illustrated in Figure 5. For all spectra, a band corresponding to the presence of adsorbed water (1,630∼1,640 cm^−1^) and hydroxide absorption bands in the range of 2,500∼3,700 cm^−1^ were observed. The band at 1,040 cm^−1^ was assigned to chloride contamination, which arises from chloride precursor used in this work. The peaks at the low wavenumbers (500∼1,000 cm^−1^) could be attributed to the SnO_2_. For the un-doped film, the peaks at 679, 784 and 968 cm^−1^ were assigned to O–Sn–O, Sn–O–Sn stretching vibrations and lattice vibrations, while the peaks at 570 and 866 cm^−1^ were due to Sn–OH bonds of the SnO_2_ crystalline phase [26–28].

The vibration frequencies of SnO_2_ were found to shift for the doped films. This shift can be ascribed to the increase in lattice disorder due to the ${\text{F}}_{\text{i}}^{-1}$ in the lattice [29]. Moreover, it is interesting to note that an addition peak near 410 cm^−1^ was observed for the films doped with NH_4_F but absent for the un-doped SnO_2_ sample. This peak could be distributed to vibration frequency of F-Sn-F [29,30]. The FTIR spectra obtained in this work were comparatively broader in comparison with previous works [26,31]. This may be due to nanocrystalline nature of our films [28]. Auger electron spectroscopy (AES) analysis of the F-doped films was also carried out in order to determine the doping level of fluorine in the films (data not shown). It was found that no fluorine was detected for all studied films *i.e.*, the concentration of the fluorine incorporated on the films in this case was less than 0.1% which is the detection limit of the analytical method. The low concentration of fluorine in the films could be due to the vaporization of fluorine from the films during the annealing-cycle process [21].

### 3.2. Gas Sensing Properties

**Figure 6.**
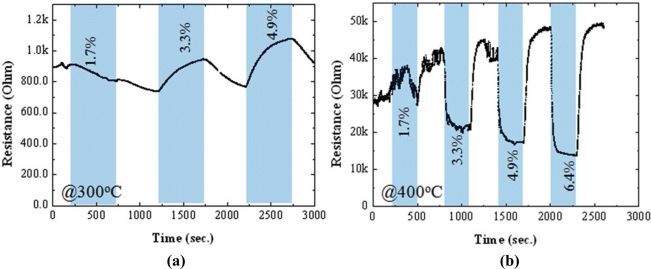
Change in resistances with respect to time of the un-doped SnO_2_ sensor in an alternating environment of N_2_ and LPG (1.7∼6.4 vol.%) at operating temperatures of **(a)** 300 °C and **(b)** 400 °C.

**Figure 7.**
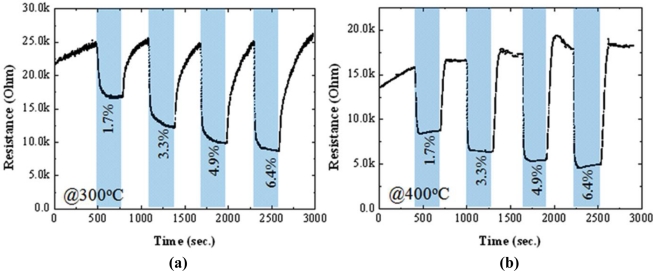
Change in resistances with respect to time of the 4.5 wt.% F-doped SnO_2_ sensor in an alternating environment of N_2_ and LPG (1.7∼6.4 vol.%) at operating temperatures of **(a)** 300 °C and **(b)** 400 °C.

In order to investigate the effect of fluorine on sensor characteristics, the resistance change of the films deposited under different concentration of NH_4_F was evaluated in the presence of LPG gases. Figures 6 and 7 present the resistance changes of the un-doped and F-doped (4.5 wt.%) sensors, respectively, at operating temperatures of 300 °C (a) and 400 °C (b). According to the Hall Effect measurements, all of the studied films were n-type in ambient temperature. The resistivity of the doped films (2∼4 × 10^−4^ Ω cm) was about two times higher than that of the un-doped one (4∼9 × 10^−4^ Ω cm), in contrast to the previous findings [13,32]. This could be due to the high proportions of ammonia fluoride to stannous chloride in this work, compared to that used in Ref. [32]. Furthermore, in N_2_ atmosphere at 300 °C, the baseline resistance of the doped sensor was also ∼2 times higher than that of the un-doped sensor, as shown in Figures 6 and 7.

An increase in resistance of SnO_2_:F may be attributed the formation of insulating fluorine compounds (SnF_x_) at grain boundaries and surfaces [33], since ‘F’ has a higher binding energy to ‘Sn’ compared to ‘O’. However, these SnF_x_ phases could not be detected by XRD analysis, probably containing very small crystallites for these phases.

A strong influence of F-doping on the gas sensitivity of SnO_2_ thin films could be found in Figures 6 and 7. At a high operating temperature (400 °C), the resistance of the un-doped sensor decreased in the presence of LPG and recovered to its baseline resistance after switching to N_2_, showing an n-type-like response to LPG [Figure 6(b)]. This is understandable because SnO_2_ sensors are known to behave as an n-type semiconductor. However, completely opposite behavior was observed for gas sensing at a low operating temperature (300 °C); the resistance of the sensor increased upon exposure to LPG, showing an p-type-like response, as displayed in Figure 6(a). It should be noted here that the transition between n-type and p-type responses could be reproducible under N_2_ atmosphere by changing the operating temperature. The p-type-like response to LPG was found in all un-doped SnO_2_ sensors only when the operating temperatures was lower than 300 °C, regardless of the film thickness (studied range: 250∼500 nm). The transition from n-type to p-type response and vice versa has been reported previously in some un-doped materials, such as In_2_O_3_ [34] and Fe_2_O_3_ [35]. The observed phenomena may be related to the small crystallite size of SnO_2_, which promotes the adsorption of catalytic water vapour [34] and/or band-bending-induced oxygen [35] at the surface of SnO2. In contrast to the un-doped sensors, F-doped sensors (Figure 7; for 4.5 wt.% of NH_4_F) showed n-type-like response, regardless of the operating temperature (studied range: 250–400 °C). As shown in Figures 6 and 7, the response of the sensors deposited both with and without F dopant increased with increasing the operating temperature. However, at a low temperature of 300 °C, F-doped sensors hold reasonably good response with high stability to LPG, compared to the un-doped one. Therefore, the temperature at 300 °C was taken as an operating temperature for further studies.

**Figure 8.**
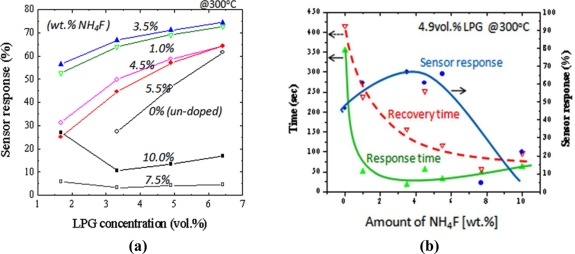
Sensing characteristics at 300 °C of the SnO_2_ films deposited at different amount of NH_4_F. **(a)** Sensor response *vs.* LPG concentration. **(b)** Sensor response time, recovery time and sensor response to 4.9 vol.% LPG.

Figure 8(a) summarizes the results of sensor response at 300 °C to N_2_-diluted LPG (1.7∼6.4 vol.%) for the sensors prepared under different amount of NH_4_F. As can be seen for this result, the enhancement in sensor response to LPG could be achieved by adding F-dopant into the SnO_2_ materials. The response increased with an increase in NH_4_F concentration. The maximum response was obtained at 3.5 wt.% of NH_4_F. However, a decrease in response was observed when the NH_4_F in the precursor was more than 5.5 wt.%. It is well known that the sensor response of the sensor increases with increasing roughness of the film, because of the increase in the number of the active adsorption sites for oxygen or hydrocarbon molecules on the sensor surfaces [36]. This could explain the maximum response of the sensor doped at 3.5 wt.% of HN_4_F. Figure 8(b) shows the sensor response time, recovery time and sensor response as a function of NH_4_F concentration. The sensors were tested under 4.9 vol.% of LPG at 300 °C. It was shown that not only the response magnitude, but the response speeds were also enhanced by increasing the NH_4_F concentration. Compared to the un-doped sensors, the response and the recovery time (0–90% of final value) of the doped sensors decreased 7 times (from ∼350 to ∼50 s) and 2.5 times (from ∼400 to ∼150 s), respectively. The enhancement of the sensing properties observed for the F-doped films could be due to the decrease in grain size and the increase in surface roughness in the resulting metal oxide films.

**Figure 9.**
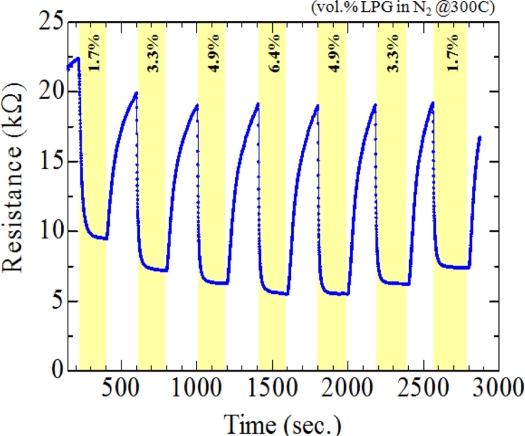
Dynamic resistance response at 300 °C of the 3.5 wt.% F-doped SnO_2_ film in an alternating environment of N2 and LPG (1.7∼6.4 vol.%).

Among of the studied sensors, the sensor deposited at 3.5 wt.% of NH_4_F showed the maximum response of ∼55% to 1.7 vol.% LPG, with a response time of ∼50 s and a recovery time of ∼150 s (Figure 9). In order to ensure correct operation in air environment, we also tested this sensor in the presence of LPG diluted with zero air.

**Figure 10.**
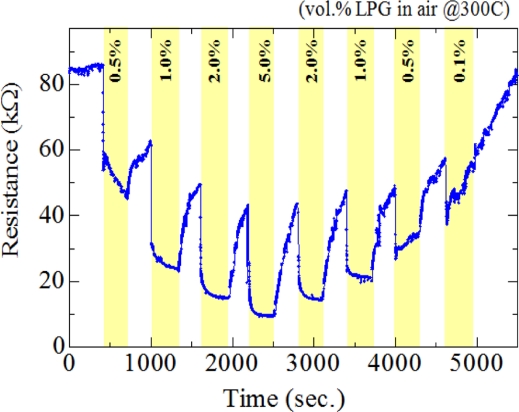
Dynamic resistance response at 300 °C of the 3.5 wt.% F-doped SnO_2_ film in an alternating environment of air and LPG (0.1∼ 5.0 vol.%).

Figure 10 shows the dynamic resistance response of the 3.5 wt.% F-doped SnO_2_ film in an alternating environment of air and LPG (0.1∼5.0 vol.%) at 300 °C. It should be noted here that the highly increase in baseline resistance under air atmosphere must be mainly caused by the adsorption of ambient oxygen on the SnO_2_ surfaces. As shown in Figure 10, the SnO_2_:F sensor could detect LPG at 0.5% by volume in air which corresponds to 25% of the LEL (lower explosive limit), showing the possibility of using this material for LPG leak detection. In order to investigate the selectivity for LPG, this sensor (3.5 wt.% F-doped SnO_2_) was tested for ethanol, methanol, acetone and LPG. The gas concentration and operating temperature in all cases were 0.5 vol.% and 300 °C, respectively. The response of the sensor to VOCs vapors was found to be slightly lower as compared to its response to LPG (Gas responses to ethanol (28%), methanol (25%), acetone (29%) and LPG (46%)), showing medium selectivity for LPG.

Since the SnO_2_ films deposited by a dip-coating technique show nanoscale textured surfaces, the sensing mechanism of the sensors may be explained by the interactions between target gases and sensor surfaces [37]. At the operating temperature of 300–400 °C atmospheric oxygen atoms are adsorbed onto the SnO_2_ surface in the term of O^−^ ions [38] by capturing electrons from the conduction band. When the SnO_2_ sensor is exposed to LPG (reducing gaseous species), LPG molecules removes adsorbed oxygen ions from the surfaces and produces water molecules along with electrons according to the following equations [39]:

$${\text{C}}_{n}{\text{H}}_{2n+2}+(3n+1){\text{O}}_{\left(\text{ads}\right)}{}^{-}\to n{\text{CO}}_{2}+(n+1){\text{H}}_{2}{\text{O}}_{\left(\text{g}\right)}+(3n+1)e^{-}$$

Here, C*_n_*H_2_*_n_*_+2_ represents a mixture of hydrocarbons like propane (C_3_H_8_; *n* = 3) and butane (C_4_H_10_; *n* = 4), the main components of LPG. This reaction produces more electrons and thus reduces the resistivity of n-type SnO_2_ and increases the resistivity of p-type SnO_2_ upon exposure to LPG. It is well known that gas response of the metal-oxide semiconductor sensors is mainly determined by the surface interactions of the target gases with the sensing material. Therefore it is certain that for the greater surface areas of the material, the interactions between the adsorbed gases and the sensor surfaces are stronger—*i.e.*, the gas response is higher [40]. In the present case, the XRD (Figure 2) and AFM (Figure 3) results show that the adding intermediate amount of fluorine into the films could decrease the crystalline size and increase the surface roughness, resulting in the formation of nanoscale-textured surfaces. An increase in surface areas [11,41] leads to more effective sites for more oxygen to be adsorbed and more interaction with LPG molecules, and, as a consequence, to enhance the sensor response (Figures 6–8). Besides, the tiny grain size of the SnO_2_:F films may become comparable to the thickness of the depletion region [10], which will also give contribution to the increased response [41]. However, further studies are needed to elucidate the gas sensing mechanism of F-doped oxides and to improve their gas sensing characteristics.

## 4. Conclusions

Tin oxide films were prepared on glass substrates by dip-coating technique using a layer-by-layer deposition cycle (alternating between dip-coating a thin layer followed by drying in air after each new layer was added). The results showed that this technique is superior to the conventional technique for both improving the film thickness uniformity and film transparency. Atomic Force Microscopy (AFM) and X-ray diffraction pattern measurements showed that the obtained thin films were nanocrystalline SnO_2_ with nanoscale-textured surfaces. The addition of fluorine to SnO_2_ was found to be advantageous for efficient detection of LPG gases, e.g., F-doped sensors are more stable at a low operating temperature (300 °C) with higher sensor response and faster response/recovery time, compared to un-doped sensor materials. Among of the studied sensors, the 3.5 wt.% F-doped SnO_2_ film showed the maximum response of ∼55% to 1.7 vol.% of LPG, with a response time of ∼50 s and a recovery time of ∼150 s. The sensor based on SnO_2_:F films could detect LPG even at a low level of 25% LEL, showing the possibility of using this material for LPG leak detection. The LPG-sensing mechanism may be explained by surface interaction between the reducing gas (LPG) and the chemisorbed oxygen ions on the SnO_2_ surfaces.

## Figures and Tables

**Table 1.** Brief review of the results on doped SnO_2_ sensors for LPG detection.

**Authors (Year) [Ref.]**	**Deposition method**	**Dopant (Doping level)**	**Test gas (Concentration)**	**Sensing performances**
M. Reddy *et al.* (1999) [4]	Electron-beam evaporation	Platinum (Pt), Palladium (Pd)	LPG (50∼800 ppm), CO, CH_4_	(Pt-SnO_2_) Response: 75% to 800 ppm LPG at 400 °C Response time: 23 s
Gupta *et al.* (2004) [5]	Magnetron sputtering and evaporation	Pd composite (7%)	LPG (0∼3,000 ppm)	Response: 65% to 3,000 ppm LPG. at 350 °C Response time: ∼10 s
Senguttuvan *et al.* (2007) [17]	Conventional solid-state route	Lead (Pb) (SnPbO_3_)	LPG (1,000 ppm)	Response: ∼48% at 150 °C
Majumder *et al.* (2008) [7]	Sputtering	Silicon (Si)	LPG (1,000∼7,000 ppm)	(Grain size: ∼90 nm) Response: 59% to 3,000 ppm LPG at 300 °C Response time: ∼30 s
Vaishampayan *et al.* (2008) [6]	Modified Pechini route	Palladium (Pd) (1.5∼3.5 mol%)	LPG (20∼1,000 ppm)	(1.5 mol% Pd, grain size: 11 nm) Response: 75∼95% at 50∼100 °C Response/Recovery time: 0.4∼0.8/3∼21 min
Thomas *et al.*(2008) [9]	Spray pyrolysis	Caesium (Cs) (0∼4 wt.%)	LPG (1,000 ppm)	(2% Cs, grain size: 18 nm) Response: 93.4% to 1,000 ppm LPG at 345 °C
Babar *et al.*(2011) [8]	Spray pyrolysis	Antimony (Sb) (0.5∼2.5 M)	LPG, Ethanol, Acetone	(grain size: 20 nm) Response: 40% to 2,000 ppm LPG at 450 °C
This work	Dip-coating	Fluoride (F) (0∼10 wt.%))	LPG (∼50,000 ppm), EtOH, MetOH, Acetone	(grain size: 4∼6 nm) Response: 46% to 5,000 ppm LPG at 300 °C
